# Colorectal cancer incidence among young adults in England: Trends by anatomical sub-site and deprivation

**DOI:** 10.1371/journal.pone.0225547

**Published:** 2019-12-05

**Authors:** Aimilia Exarchakou, Liam J. Donaldson, Fabio Girardi, Michel P. Coleman

**Affiliations:** 1 Cancer Survival Group, Non-Communicable Disease Epidemiology Department, London School of Hygiene and Tropical Medicine, London, United Kingdom; 2 Non-Communicable Disease Epidemiology Department, London School of Hygiene and Tropical Medicine, London, United Kingdom; Universita degli Studi di Ferrara, ITALY

## Abstract

**Background:**

Colorectal cancer incidence in the UK and other high-income countries has been increasing rapidly among young adults. This is the first analysis of colorectal cancer incidence trends by sub-site and socioeconomic deprivation in young adults in a European country.

**Methods:**

We examined age-specific national trends in colorectal cancer incidence among all adults (20–99 years) diagnosed during 1971–2014, using Joinpoint regression to analyse data from the population-based cancer registry for England. We fitted a generalised linear model to the incidence rates, with a maximum of two knots. We present the annual percentage change in incidence rates in up to three successive calendar periods, by sex, age, deprivation and anatomical sub-site.

**Results:**

Annual incidence rates among the youngest adults (20–39 years) fell slightly between 1971 and the early 1990s, but increased rapidly from then onwards. Incidence Rates (IR) among adults 20–29 years rose from 0.8 per 100,000 in 1993 to 2.8 per 100,000 in 2014, an average annual increase of 8%. An annual increase of 8.1% was observed for adults aged 30–39 years during 2005–2014. Among the two youngest age groups (20–39 years), the average annual increase for the right colon was 5.2% between 1991 and 2010, rising to 19.4% per year between 2010 (IR = 1.2) and 2014 (IR = 2.5). The large increase in incidence rates for cancers of the right colon since 2010 were more marked among the most affluent young adults. Smaller but substantial increases were observed for cancers of the left colon and rectum. Incidence rates in those aged 50 years and older remained stable or decreased over the same periods.

**Conclusions:**

Despite the overall stabilising trend of colorectal cancer incidence in England, incidence rates have increased rapidly among young adults (aged 20–39 years). Changes in the prevalence of obesity and other risk factors may have affected the young population but more research is needed on the cause of the observed birth cohort effect. Extension of mass screening may not be justifiable due to the low number of newly diagnosed cases but clinicians should be alert to this trend.

## Introduction

Colorectal cancer is one of the commonest cancers worldwide. In 2016, it was the third most frequent cancer among men and women in England, accounting for 12% of all new cancer cases [1]. It is predominantly a disease of older people, with the highest incidence rates amongst adults aged 70 years and over [2].

In several high-income countries, including the United States, Australia and Canada, incidence of invasive colorectal cancer has declined in older men and women, partly because of screening programmes for colorectal cancer [3–5], which enable the detection and removal of pre-invasive polyps. In the United Kingdom, the National Health Service (NHS), introduced population-based screening in 2006 for all people aged 60 years and over, using the faecal occult blood test.

Recent population-based studies have shown that colorectal cancer incidence has been increasing in age groups not currently targeted by screening programmes, especially adolescents and young adults [6–10]. In the United States, colorectal cancer incidence in the under-50s is higher now than it was in the mid-1990s, with marked differences between the sexes and between racial and ethnic groups [11–13]. In the United Kingdom, age-standardised incidence rates have remained stable, but rates in adults aged 20–39 have accelerated rapidly in the last 25 years [9].

Socioeconomic variation in colorectal cancer incidence in the UK, has shifted. In the 1980s, affluence carried a higher risk of colorectal cancer, but during the 1990s, surveillance data pointed to an increased risk for adults, especially men, in areas of higher deprivation. Since then, in the period 1996 to 2010, an association has emerged between colorectal cancer incidence and deprivation in men, but not in women [14, 15]. However, a deprivation gradient in women became apparent in 2010–2012 [16].

In this study, we describe colorectal cancer incidence trends among young adults in England over a 44-year period. We also examine differences in these trends by anatomical sub-site and between socioeconomic groups.

## Methods

### Study design and participants

This is a longitudinal study, analysing trends in population incidence rates of colorectal cancer over the period 1971–2014 in England.

We obtained anonymised individual records from the National Cancer Registry for England, held by the Office for National Statistics (ONS) for persons diagnosed with colorectal cancer between 1971 and 2014.

ONS uses standardised procedures to ensure high-quality data. We applied additional checks to identify and exclude incomplete, ineligible or inconsistent tumour records [17], as well as records of a second primary tumour in the colon or rectum in the same person. We excluded less than 5% of all records, leaving 1,073,624 adults aged 20–99 years diagnosed with a primary, invasive malignancy of the large bowel during 1971–2014. We grouped cancers into anatomical sub-sites: left colon (153.2–154.0, C18.5-C18.7), right colon (153.0–153.6, C18.0-C18.4), rectum (154.1, C19-C20) and unspecified tumours of the colon (153.8–153.9, C18.8-C18.9) [18–20].

We examined overall incidence trends for seven age groups (20–29, 30–39, 40–49, 50–59, 60–69, 70–79, 80–99 years), chosen to enable comparisons with studies in the United States and Australia [8, 21]. We further analysed incidence trends by deprivation and anatomical sub-site for young adults, aged 20–39 years.

We obtained information on deprivation from the Index of Multiple Deprivation (IMD 2015) [22], an ecological measure based on scores in seven distinct domains of deprivation assigned to each of the so-called Lower-Layer Super Output Areas (LSOAs). LSOAs comprise 32,844 small administrative areas that cover the whole of England. They are designed to be relatively homogeneous for a range of socio-economic variables, with an average population of only 1,500 [23]. IMD 2015 uses the geographic boundaries of LSOAs, as revised following the 2011 Census. IMD scores for each domain can be used individually or combined for a summary measure of deprivation. For this study, we used the combined IMD scores, ranked in five quintiles. These were defined by the national distribution of LSOA scores in seven domains: Income, Employment, Education, Health and Disability, Crime, Barriers to Housing and Services and Living Environment Deprivation. Patients were assigned to one of five deprivation levels from 1 (“least deprived”, or “most affluent”) to 5 (“most deprived”) according to the patient’s postcode of residence at the time of diagnosis.

The number of colorectal cancer registrations between years 1971 and 2014, by year, sex and age group were applied to the corresponding populations from ONS to obtain the annual incidence rates [24].

Incidence trends by sub-site and deprivation level were focused on the most recent period (2001–2014), because population counts by IMD 2015 deprivation score have only been available since 2001 [25].

### Ethical approval

The data used in this study were analysed under approvals from the UK’s statutory Health Research Authority (PIAG 1-05(c)/2007; ECC 1-05(a)2010) and NHS Research Ethics Committee (13/LO/0610).

### Statistical analyses

We used the Joinpoint Regression Program from the US National Cancer Institute [26] to analyse trends in the incidence rate. This methodology fits the simplest linear trend to the data in a given calendar period. The linear trends within successive calendar periods are joined at knots or “joinpoints”, which mark statistically significant changes (increase or decrease) in the slope. The location (calendar year) of the knots is determined by a Monte Carlo permutation test with a two-sided statistical significance of α = 5%. This gives a piecewise linear trend over the entire period covered by the data.

We fitted a generalised linear model (log-linear) to the incidence rates, with a maximum of two knots, allowing us to describe trends in the incidence rate as the annual percentage change (APC) during up to three successive calendar periods. The knots, and therefore the length of the calendar periods, may differ between analyses. The maximum length of the incidence trends was between 1971 and 2014 for the rates by sex, age group and sub-site and between 2001 and 2014 for the rates by sub-site and deprivation. For the age-standardised rates we used the European Standard Population weights, modified to reflect only the adult population (15–99 years).

## Results

### Incidence by age group

During 1971–2014, 1,073,624 people aged between 20–99 years were diagnosed with colorectal cancer in England (Table 1 and S1 Table), of whom 562,833 were males (52.4%) and 510,791 females (47.5%).

**Table 1:** Annual Percentage Change (APC, %) in colorectal cancer incidence rates by sex, age and calendar period (segment) of diagnosis: England, 1971–2014.

	Persons* (N = 1,073,624)					Males* (N = 562,833)					Females* N = 510,791)				
Age (years)	Segment	APC (%)	95% CI			Segment	APC (%)	95% CI			Segment	APC (%)	95% CI		
**20–29**	**1971–1993**	-1.5	-2.6	to	-0.5	**1971–1994**	-0.7	-1.9	to	0.6	**1971–1993**	-2.2	-3.6	to	-0.7
	**1993–2014**	8.0	7.1	to	8.8	**1994–2014**	7.3	6.1	to	8.4	**1993–2014**	8.9	7.8	to	10.1
**30–39**	**1971–1990**	-1.7	-2.4	to	-1.0	**1971–1990**	-1.4	-2.3	to	-0.6	**1971–1994**	-1.8	-2.4	to	-1.1
	**1990–2005**	1.2	0.0	to	2.3	**1990–2006**	1.2	0.0	to	2.3	**1994–2009**	3.0	1.7	to	4.3
	**2005–2014**	8.1	6.2	to	10.0	**2006–2014**	8.4	6.3	to	10.6	**2009–2014**	12.0	6.5	to	17.7
**40–49**	**1971–2003**	-0.3	-0.4	to	-0.1	**1971–2008**	0.0	-0.2	to	0.2	**1971–2003**	-0.5	-0.7	to	-0.3
	**2003–2014**	1.5	0.7	to	2.3	**2008–2014**	1.9	0.1	to	3.8	**2003–2014**	1.9	1.0	to	2.8
**50–59**	**1971–1993**	0.7	0.5	to	0.9	**1971–1995**	1.2	1.0	to	1.5	**1971–2009**	0.0	-0.1	to	0.1
	**1993–2014**	0.1	-0.1	to	0.3	**1995–2014**	0.0	-0.3	to	0.3	**2009–2014**	2.0	0.1	to	3.9
**60–69**	**1971–2011**	1.1	1.0	to	1.2	**1971–1996**	1.7	1.4	to	2.0	**1971–2011**	0.6	0.5	to	0.7
	**2011–2014**	-4.7	-7.8	to	-1.5	**1996–2011**	0.8	0.3	to	1.3	**2011–2014**	-4.0	-8.0	to	0.2
	** **					**2011–2014**	-4.9	-9.8	to	0.2	** **				
**70–79**	**1971–1987**	0.7	0.4	to	1.1	**1971–1987**	0.8	0.4	to	1.2	**1971–2011**	0.8	0.7	to	0.9
	**1987–2000**	1.9	1.4	to	2.3	**1987–2000**	2.1	1.6	to	2.7	**2011–2014**	-3.1	-6.0	to	0.0
	**2000–2014**	0.1	-0.2	to	0.4	**2000–2014**	-0.2	-0.5	to	0.2	** **				
**80–99**	**1971–2014**	0.9	0.8	to	1.0	**1971–2009**	1.1	1.1	to	1.2	**1971–2014**	0.6	0.6	to	0.7
						**2009–2014**	-0.4	-1.7	to	0.9	** **				

* Diagnosed with colorectal cancer

The age distribution is shifted to older ages with 81.3% of cases aged over 60 years old. Only 3,148 colorectal cases (0.2%) were aged 20–29 years and 11,643 (1.0%) aged 30–39 years (S1 Table).

Age-standardised incidence rates in males increased by 1.3% (95% confidence interval (CI): 1.1–1.4%) per year between 1971 and 1998 but stabilised during 1998–2012 and decreased by 3.4% (95% CI: -6.3- -0.5%) per year during 2012–2014 (S1 Fig). Rates among females remained relatively stable in 1971–2014 (0.6% per year; 95% CI: 0.5–0.6%).

Colorectal cancer incidence rates in young adults (aged 20–39 years) remained 40–180 times lower than in persons aged 60–69 years during 1971–2014 (S2 Table). Among those aged 20–29 years, the incidence rate was 0.7 per 100,000 in 1971 and fell by an average 1.5% per year during 1971–1993. The decline was similar in 30-39-year-olds (1.7% per year; 1971–1990) (Table 1). Among those aged 40–49 years, the decrease in incidence rates was smaller (0.3% per year, 1971–2003).

From the early 1990s, incidence rates increased substantially among adults aged 20–39 years.

For the youngest age group (20–29 years), the average annual increase was 8% (95% CI: 7.1–8.8%) per year, rising from 0.8 per 100,000 in 1993 to 2.8 per 100,000 in 2014 (S2 Table). For those aged 30–39 years, the annual increase after 2005 was 8.1% (95% CI: 6.2–10.0%) per year. In 2005, the incidence rate was 3.9 per 100,000, rising to 7.6 per 100,000 by 2014. We found a smaller annual increase in adults aged 40–49 years during 2003–2014 (1.5% per year).

In adults over 50 years of age, incidence rates remained stable or increased less rapidly, around 1% per year. For those aged 60–69 years, incidence rates actually fell by 4.7% per year from 2011 to 2014.

### Incidence trends among young adults (20–39 years)

#### Anatomical sub-site

**Fig 1:**
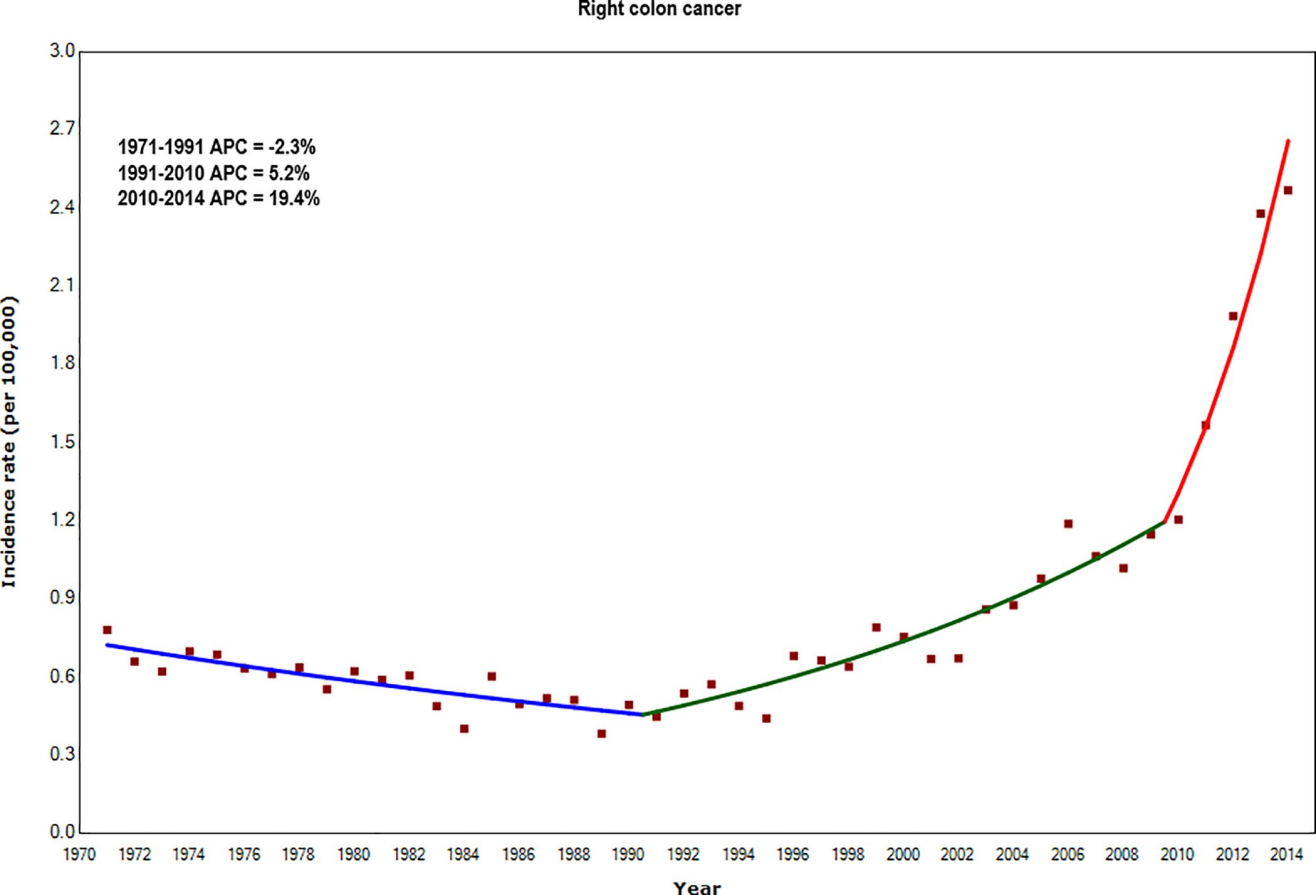
Annual Percentage Change (APC) in incidence rates of right colon cancer for adults aged 20–39 years: England 1971–2014.

The increasing trend in colorectal cancer incidence in adults aged 20–39 years was more marked for cancers of the right colon than left colon and rectum. Incidence rates increased by an average 5.2% per year from 0.5 per 100,000 in 1991 to 1.2 per 100,000 in 2010. During 2010–2014, the rate of the increase accelerated sharply to 19.4% (95% CI: 14.5–24.6%) per year (Fig 1 and S3 Table), with the rate rising to 2.5 per 100,000 in 2014.

**Fig 2:**
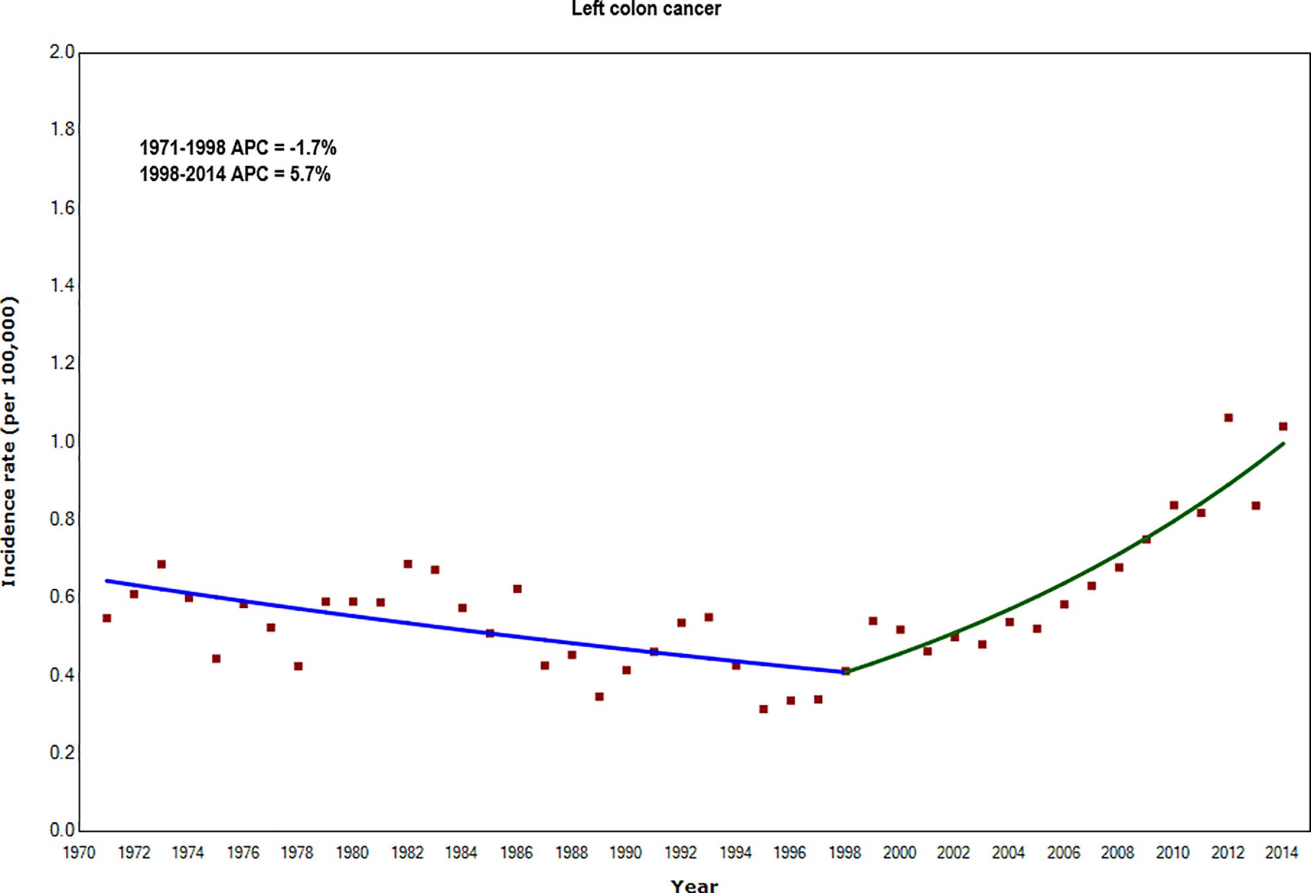
Annual Percentage Change (APC) in incidence rates of left colon cancer for adults aged 20–39 years: England 1971–2014.

For left-sided colon cancer, incidence rates increased less rapidly, by 5.7% per year during 1998–2014 (Fig 2 and S3 Table).

**Fig 3:**
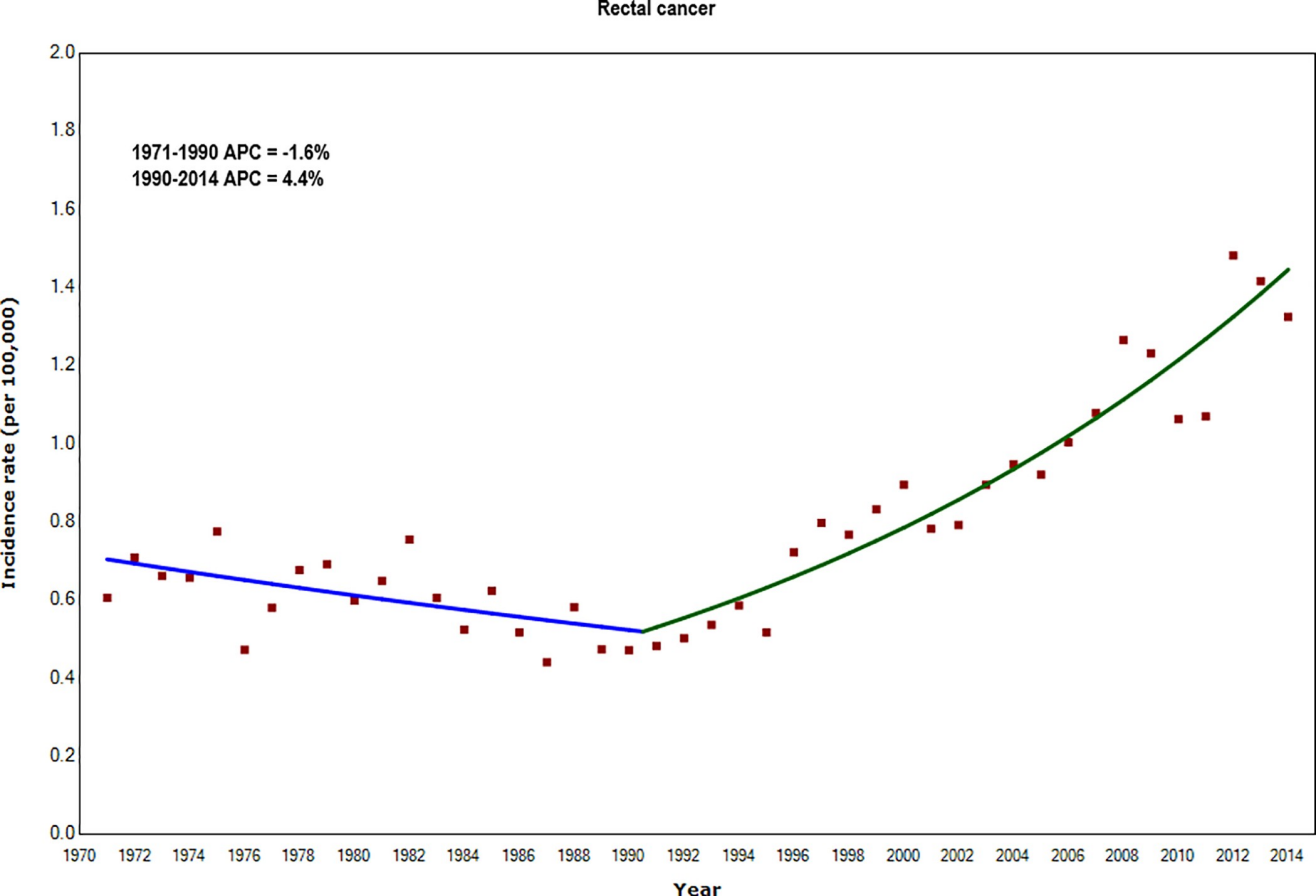
Annual Percentage Change (APC) in incidence rates of rectal cancer for adults aged 20–39 years: England 1971–2014.

Incidence rates for rectal cancer also increased in younger adults, by an average of 4.4% per year during 1990–2014 (Fig 3 and S3 Table). In contrast, incidence rates of colon tumours with unspecified sub-site fell by an average 3.2% per year between 1996 and 2014 (S3 Table).

#### Socio-economic deprivation (2001–2014)

Incidence rates in the two youngest age groups (20–39 years) were generally higher in the more deprived groups than in the less deprived (Table 2).

**Table 2:** Annual Percentage Change (APC) in colorectal cancer incidence rates by anatomical sub-site and deprivation for adults aged 20–39: England, 2001–2014.

Anatomical sub-site		Crude incidence rate		Trends in incidence				
		2001	2014	Segments	APC (%)	95% CI		
**Right colon**	**Most affluent**	**0.49**	**2.90**	**2001–2010**	4.7	- 0.4	to	10.1
** **				**2010–2014**	25.2	12.0	to	39.9
** **	**2**	**0.79**	**1.80**	**2001–2014**	9.8	6.3	to	13.4
** **	**3**	**0.46**	**2.17**	**2001–2014**	11.9	8.7	to	15.2
** **	**4**	**0.65**	**2.70**	**2001–2014**	11.4	8.7	to	14.2
** **	**Most deprived**	**0.92**	**2.71**	**2001–2014**	10.0	6.9	to	13.1
**Left colon**	**Most affluent**	**0.44**	**1.33**	**2001–2014**	7.8	3.7	to	12.0
** **	**2**	**0.49**	**1.02**	**2001–2014**	5.5	2.4	to	8.6
** **	**3**	**0.46**	**1.10**	**2001–2014**	8.6	6.0	to	11.3
** **	**4**	**0.36**	**1.07**	**2001–2014**	8.7	6.5	to	10.9
** **	**Most deprived**	**0.56**	**0.77**	**2001–2014**	4.6	2.0	to	7.3
**Rectum**	**Most affluent**	**0.73**	**1.24**	**2001–2014**	6.1	2.0	to	10.5
** **	**2**	**0.86**	**1.57**	**2001–2014**	4.5	1.9	to	7.2
** **	**3**	**0.92**	**1.35**	**2001–2014**	3.3	- 0.6	to	7.3
** **	**4**	**0.62**	**1.28**	**2001–2014**	4.8	1.7	to	8.0
** **	**Most deprived**	**0.79**	**1.22**	**2001–2014**	3.8	0.6	to	7.2
**Colon, unspecified**	**Most affluent**	**0.20**	**0.23**	**2001–2014**	2.4	- 1.6	to	6.6
** **	**2**	**0.41**	**0.12**	**2001–2014**	- 1.0	- 8.3	to	6.8
** **	**3**	**0.32**	**0.17**	**2001–2014**	- 7.6	- 11.9	to	- 3.1
** **	**4**	**0.13**	**0.03**	**2001–2014**	3.4	- 2.4	to	9.5
** **	**Most deprived**	**0.30**	**0.15**	**2001–2014**	- 7.7	- 11.5	to	- 3.8
**All sub-sites**	**Most affluent**	**1.86**	**5.70**	**2001–2014**	7.7	5.6	to	9.8
** **	**2**	**2.55**	**4.51**	**2001–2014**	6.1	4.1	to	8.1
** **	**3**	**2.16**	**4.79**	**2001–2014**	6.9	5.1	to	8.7
** **	**4**	**1.76**	**5.07**	**2001–2014**	8.0	7.2	to	8.8
** **	**Most deprived**	**2.57**	**4.86**	**2001–2014**	5.4	4.0	to	7.0

Whilst incidence rates increased consistently in all deprivation groups and at each anatomical sub-site, the increasing trend for cancers of the right colon accelerated sharply in the most affluent group, from 4.7% per year during 2001–2010 to 25.2% (95% CI: 12–39.9%) per year during 2010–2014. Crude incidence rates increased from 0.5 per 100,000 in 2001 to 3.0 per 100,000 in 2014. The rates of increase in the other deprivation groups were smaller, but still substantial, at 10–12% per year (Table 2).

## Discussion

To our knowledge, this is the first study to examine incidence trends for colorectal cancer in relation to socio-economic deprivation and anatomical site in the younger population of England.

Whilst age-standardised colorectal cancer incidence rate in England has stabilised over the last decade, we found a sharp increase in the rate for young adults. This observation is in line with studies in Europe, the United States, Canada and Australia [6, 8, 9, 21, 27]. It is striking that incidence rates amongst young men and women aged 20–29 years and 30–39 years almost tripled between 1990 and 2014. Increases in incidence among men and women aged 40–49 years were much smaller. Rates in older age groups either remained stable or slightly decreased.

The rise in colorectal cancer incidence rates in England started after 1993 among 20-29-year-olds and after 2005 among 30-39-year-olds suggesting a cohort effect. In the preceding three decades, risk factors such as overweight and obesity had become more prevalent [28]. These risk factors would need to have acted early in life, probably childhood or adolescence, to account for an increase in colorectal cancer risk mainly restricted to young adults [29]. In England, overweight and obesity among children aged 2–15 years rose from 25% in 1995 to 30% in 2017 [30]. The prevalence of obesity is also related to the level of physical activity in the population. Activity levels in children 5–15 years old have dropped by 20% since the 1960s, and in 2017, only 18% of children and young people met the Chief Medical Officer’s current guidelines of at least 60 minutes of exercise per day [30].

In the International Agency for Research on Cancer 2018 monograph, consumption of red meat was classified as “probably carcinogenic” and processed meat as “carcinogenic” [31]. Alcohol use and reduced consumption of dietary fibre are also associated with an increased risk of colorectal cancer [32]. Adolescents and young adults have been acquiring less of their energy intake at home and more at restaurants and fast-food outlets [33, 34], increasing their exposure to a poor-quality and potentially carcinogenic diet. However, evidence on the consumption of specific foods or nutrients during early life and the risk of colorectal cancer later in life is sparse and conflicting [35].

In contrast to the US studies [5, 21], we found that for young adults (20–39 years) the increases in incidence were more marked for cancers of the right colon, especially since 2010. Given that distribution of risk factors in the UK and the US are similar [36], we hypothesise that the shift to an increase in right-sided colon cancers in the UK could be due to different referral patterns and clinical management. The UK National Institute for Health and Care Excellence (NICE) guidelines do not recommend endoscopy or imaging for patients with IBS (Irritable Bowel Syndrome). By contrast, 45% of 200,000 US patients with IBS received an endoscopy during 2001–12, and 36% received at least three gastrointestinal medical procedures [37], suggesting that sub-clinical, early-stage tumours of the rectum and left colon might be detected more often.

Incidence rates in young adults increased for all deprivation groups and anatomical sub-sites. However, the increase in incidence rates for right-sided colon cancer was largely attributable to the trend in the most affluent persons: the sharp increases coincided in time and were similar in magnitude. Common risk factors such as obesity and diabetes are mainly associated with deprivation [38–40], but colorectal cancer screening uptake [41] and other health-seeking behaviours are more common among affluent groups, which could lead to a more timely diagnosis [42]. Clinical suspicion for colorectal cancer among young adults is generally low, while greater awareness of symptoms and better navigation of the health system could help explain the excess of right-sided colon cancer among the most affluent.

We have described an increasing incidence of colorectal cancer in young adults at the population level, but colorectal cancer in this age group remains uncommon, and clinical suspicion for colorectal cancer in a young adult will inevitably be lower than in an older adult. Nevertheless, red-flag symptoms in a younger person such as persistent changes in bowel function, rectal bleeding or abdominal pain should not be lightly dismissed as being unlikely to stem from a serious cause. It is important that clinical guidelines for colorectal cancer and postgraduate education curricula address this need, to enable primary care physicians to identify the right patients for referral, and to avoid additional strain on health service diagnostic resources.

The United Kingdom’s national screening programme for colorectal cancer targets adults aged 60 years and over. Extension of mass screening to much younger age groups is not likely to be justifiable in public health terms [43] and is unlikely to become policy. However, if it were possible to delineate high-risk groups of young adults with sufficient precision, identifying symptomatic individuals for targeted colonoscopy could prove beneficial in preventing invasive malignancy or promptly diagnosing any invasive colorectal cancer.

In 2011, in the United Kingdom, the “*Be Clear on Cancer*” campaign [44] was launched to raise awareness of colorectal cancer symptoms and signs at a regional and national level. It was particularly successful in fighting the embarrassment associated with alarming symptoms [45]. However, it was specifically aimed at people aged over 55 years, and the message may have been less relevant to the younger population. Increasing awareness of the symptoms and signs of colorectal cancer among both the general population of younger adults and clinicians could be beneficial. Online symptom-checkers for the public and for primary care physicians could also be helpful [46].

### Limitations

In 14% of colorectal cancers diagnosed during 1971–1990, the anatomical sub-site was not specified, but this proportion fell to 6% during 2003–2014 (S1 Table), probably due to better pathological reporting and improved cancer registration and coding. The bias introduced by any misclassification affected all age groups, hence it is unlikely to explain the observed trends.

The Index of Multiple Deprivation (IMD) is an ecological measure based on the characteristics of the area in which each individual is resident, but socio-economic heterogeneity among individuals in the same category of the IMD would bias any differences towards the null. This means that the differences in incidence trends between the five socio-economic levels we report here are likely to be an underestimation of the true differences.

## Conclusion

More detailed studies are warranted to investigate the interplay between behavioural and environmental risk factors and its impact on the rapidly increasing incidence of colorectal cancer in young adults.

Despite the magnitude of the increase in incidence rates among young adults 20–39 years, the number of newly diagnosed cases remains far lower than in adults aged 50 years or more. Incidence rates in the over-50s decreased or barely changed. However, if the trend we report in this recent birth cohort were to continue unchecked, it would foreshadow a very substantial increase in the number of older adults being diagnosed with colorectal cancer over the next 20–30 years.

## Supporting information

S1 Table Characteristics of colorectal cancer patients in three calendar periods of diagnosis: England, 1971–2014. (DOCX)

S2 Table Annual incidence rates of colorectal cancer (per 100,000) by age group: England, 1971–2014. (DOCX)

S3 Table Annual Percentage Change (APC) in colorectal cancer incidence rates by anatomical sub-site and calendar period of diagnosis in adults aged 20–39 years: England, 1971–2014. (DOCX)

S1 Fig Annual Percentage Change (APC) in age-standardised colorectal cancer incidence rates (adults): England, 1971–2014. (TIFF)
