# Supplementary material for: Colorectal cancer incidence among young adults in England: Trends by anatomical sub-site and deprivation
Source: PLoS One. 2019 Dec 5;14(12):e0225547. doi: 10.1371/journal.pone.0225547 (PMC6894790; doi:10.1371/journal.pone.0225547)
Supplement: S1 Table — (DOCX) [file pone.0225547.s001.docx]

**S1 Table. Characteristics of colorectal cancer patients in three calendar periods of diagnosis: England, 1971-2014**

|  | **1971-1990** | | | **1991-2002** | | | **2003-2014** | | |
| --- | --- | --- | --- | --- | --- | --- | --- | --- | --- |
|  | **N** | **%** | **%** | **N** | **%** | **%** | **N** | **%** | **%** |
| **Sex** |  |  |  |  |  |  |  |  |  |
| Men | 191,002 | 48.9 |  | 163,947 | 53.1 |  | 207,884 | 56 |  |
| Women | 199,648 | 51.1 |  | 144,990 | 46.9 |  | 166,153 | 44 |  |
| **Age (years)** |  |  |  |  |  |  |  |  |  |
| 20-29 | 911 | 0.2 |  | 604 | 0.2 |  | 1,633 | 0.4 |  |
| 30-39 | 4,445 | 1.1 |  | 2,893 | 0.9 |  | 4,305 | 1.2 |  |
| 40-49 | 16,724 | 4.3 |  | 11,603 | 3.8 |  | 14,023 | 3.7 |  |
| 50-59 | 51,265 | 13.1 |  | 35,527 | 11.5 |  | 41,261 | 11.0 |  |
| 60-69 | 107,654 | 27.6 |  | 75,938 | 24.6 |  | 92,291 | 25 |  |
| 70-79 | 133,638 | 34.2 |  | 106,433 | 34.5 |  | 118,764 | 32 |  |
| 80-99 | 76,013 | 19.5 |  | 75,939 | 24.6 |  | 101,760 | 27 |  |
| **Anatomical subsite** |  |  |  |  |  |  |  |  |  |
| **Right colon** |  |  |  |  |  |  |  |  |  |
| 153.0 (ICD-9) | 26,105 | 28.3 |  | 1,344 | 1.8 |  |  |  |  |
| 153.1 (ICD-9) | 23,728 | 25.8 |  | 3,680 | 4.9 |  |  |  |  |
| 153.4 (ICD-9) | 29,736 | 32.3 |  | 12,028 | 15.9 |  |  |  |  |
| 153.5 (ICD-9) | 772 | 0.8 |  | 375 | 0.5 |  |  |  |  |
| 153.6 (ICD-9) | 11,745 | 12.8 |  | 4,489 | 5.9 |  |  |  |  |
| C18.0 (ICD-10) |  |  |  | 28,692 | 38.0 |  | 53,225 | 46 |  |
| C18.1 (ICD-10) |  |  |  | 1,162 | 1.5 |  | 4,490 | 3.9 |  |
| C18.2 (ICD-10) |  |  |  | 11,380 | 15.1 |  | 29,650 | 26 |  |
| C18.3 (ICD-10) |  |  |  | 3,835 | 5.1 |  | 10,521 | 9.0 |  |
| C18.4 (ICD-10) |  |  |  | 8,500 | 11.3 |  | 18,429 | 16 |  |
| ***Right Colon total*** | *92,086* | *100* |  | *75,485* | *100* |  | *116,315* | *100* |  |
| ***Right colon % of colorectal*** |  |  | *24%* |  |  | *24%* |  |  | *28%* |
| **Left colon** |  |  |  |  |  |  |  |  |  |
| 153.2 (ICD-9) | 13,585 | 12.0 |  | 2,439 | 3.1 |  |  |  |  |
| 153.3 (ICD-9) | 71,734 | 63.6 |  | 17,617 | 22.6 |  |  |  |  |
| 153.7 (ICD-9) | 5,310 | 4.7 |  | 1,673 | 2.1 |  |  |  |  |
| 154.0 (ICD-9) | 22,243 | 19.7 |  | 6,750 | 8.6 |  |  |  |  |
| C18.5 (ICD-10) |  |  |  | 3,901 | 5.0 |  | 8,002 | 6.0 |  |
| C18.6 (ICD-10) |  |  |  | 5,268 | 6.7 |  | 10,577 | 7.9 |  |
| C18.7 (ICD-10) |  |  |  | 40,467 | 51.8 |  | 78,709 | 58.6 |  |
| ***Left Colon Total*** | *112,872* | *100* |  | *78,115* | *100* |  | *134,311* | *100* |  |
| ***Left colon % of colorectal*** |  |  | *29%* |  |  | *25%* |  |  | *33%* |
| **Rectum** |  |  |  |  |  |  |  |  |  |
| 1541 (ICD-9) | 130,520 | 100 |  | 28,871 | 26.7 |  |  |  |  |
| C19.0 (ICD-10) |  |  |  | 16,406 | 15.2 |  | 27,224 | 20 |  |
| C20.0 (ICD-10) |  |  |  | 62,874 | 58.1 |  | 107,087 | 80 |  |
| ***Rectum total*** | *130,520* | *100* |  | *108,151* | *100* |  | *134,311* | *100* |  |
| ***Rectum % of colorectal*** |  |  | *33%* |  |  | *35%* |  |  | *33%* |
| **Colon, unspecified** |  |  |  |  |  |  |  |  |  |
| 153.8 (ICD-9) | 19,445 | 35.2 |  | 470 | 1.0 |  |  |  |  |
| 153.9 (ICD-9) | 35,727 | 64.8 |  | 17,021 | 36.1 |  |  |  |  |
| C18.8 (ICD-10) |  |  |  | 955 | 2.0 |  | 1,007 | 3.9 |  |
| C18.9 (ICD-10) |  |  |  | 28,740 | 60.9 |  | 25,116 | 96 |  |
| ***Colon, unspecified total*** | *55,172* | *100* |  | *47,186* | *100* |  | *26,123* | *100* |  |
| ***Colon, unspecified % of colorectal*** |  |  | *14%* |  |  | *15%* |  |  | *6%* |
