# Supplementary material for: Colorectal cancer incidence among young adults in England: Trends by anatomical sub-site and deprivation
Source: PLoS One. 2019 Dec 5;14(12):e0225547. doi: 10.1371/journal.pone.0225547 (PMC6894790; doi:10.1371/journal.pone.0225547)
Supplement: S2 Table — (DOCX) [file pone.0225547.s002.docx]

**S2 Table. Annual incidence rates of colorectal cancer (per 100,000) by age group: England, 1971-2014**

|  | **Age groups** | | | | | | |
| --- | --- | --- | --- | --- | --- | --- | --- |
| **Year** | **20-29** | **30-39** | **40-49** | **50-59** | **60-69** | **70-79** | **80-99** |
| **1971** | 0.7 | 4.4 | 16.5 | 49.4 | 117.6 | 234.2 | 321.7 |
| **1972** | 0.8 | 4.2 | 15.9 | 46.4 | 119.8 | 227.0 | 317.3 |
| **1973** | 0.6 | 4.2 | 15.9 | 48.5 | 119.3 | 243.7 | 355.1 |
| **1974** | 1.1 | 3.9 | 16.5 | 49.1 | 121.2 | 240.4 | 319.2 |
| **1975** | 0.8 | 4.0 | 17.2 | 48.9 | 123.8 | 253.9 | 320.0 |
| **1976** | 0.5 | 4.0 | 16.4 | 52.4 | 125.1 | 241.8 | 325.6 |
| **1977** | 0.7 | 3.4 | 14.3 | 44.4 | 114.6 | 223.6 | 309.3 |
| **1978** | 0.7 | 3.4 | 13.9 | 49.8 | 121.8 | 238.3 | 327.4 |
| **1979** | 0.6 | 3.9 | 16.8 | 55.1 | 129.3 | 249.1 | 342.2 |
| **1980** | 0.6 | 3.6 | 14.8 | 53.1 | 130.0 | 260.5 | 372.0 |
| **1981** | 0.6 | 3.6 | 18.0 | 53.5 | 129.2 | 254.7 | 372.0 |
| **1982** | 0.8 | 4.2 | 15.5 | 55.4 | 130.5 | 254.8 | 344.7 |
| **1983** | 0.7 | 3.6 | 16.9 | 52.8 | 132.7 | 256.8 | 382.1 |
| **1984** | 0.5 | 3.2 | 15.3 | 56.5 | 135.9 | 257.1 | 359.7 |
| **1985** | 0.7 | 3.6 | 14.8 | 56.3 | 136.6 | 262.4 | 371.0 |
| **1986** | 0.7 | 3.4 | 15.3 | 53.7 | 133.4 | 260.5 | 363.8 |
| **1987** | 0.7 | 3.1 | 15.7 | 53.8 | 133.2 | 251.5 | 360.2 |
| **1988** | 0.7 | 3.8 | 15.1 | 56.2 | 143.0 | 269.3 | 370.7 |
| **1989** | 0.6 | 3.0 | 15.7 | 56.7 | 153.8 | 284.2 | 384.4 |
| **1990** | 0.7 | 3.1 | 15.6 | 61.0 | 150.5 | 290.2 | 398.0 |
| **1991** | 0.5 | 3.3 | 15.5 | 60.5 | 159.2 | 278.9 | 396.0 |
| **1992** | 0.7 | 3.4 | 16.8 | 64.6 | 165.7 | 294.7 | 407.7 |
| **1993** | 0.8 | 3.8 | 16.3 | 63.9 | 173.0 | 304.3 | 414.7 |
| **1994** | 0.6 | 3.3 | 16.0 | 60.2 | 166.1 | 304.0 | 412.1 |
| **1995** | 0.5 | 2.9 | 14.9 | 63.3 | 167.1 | 298.6 | 413.0 |
| **1996** | 1.0 | 3.4 | 17.4 | 63.0 | 181.7 | 317.0 | 434.3 |
| **1997** | 0.7 | 3.7 | 17.4 | 62.3 | 178.3 | 329.3 | 424.0 |
| **1998** | 1.1 | 2.9 | 16.3 | 62.8 | 181.6 | 336.7 | 413.7 |
| **1999** | 1.0 | 4.0 | 16.6 | 63.0 | 176.0 | 340.0 | 439.5 |
| **2000** | 0.7 | 3.9 | 15.0 | 63.5 | 180.5 | 351.3 | 439.8 |
| **2001** | 1.0 | 3.5 | 14.7 | 62.0 | 171.3 | 332.7 | 441.1 |
| **2002** | 0.9 | 3.1 | 16.0 | 61.4 | 173.2 | 331.7 | 445.2 |
| **2003** | 1.4 | 3.7 | 15.0 | 60.7 | 174.2 | 332.3 | 440.2 |
| **2004** | 1.2 | 4.2 | 15.8 | 62.1 | 175.2 | 344.2 | 456.7 |
| **2005** | 1.6 | 3.9 | 16.4 | 66.2 | 167.9 | 348.9 | 453.0 |
| **2006** | 1.7 | 4.2 | 16.6 | 66.3 | 174.1 | 346.9 | 457.7 |
| **2007** | 1.6 | 4.2 | 15.5 | 61.0 | 181.5 | 345.7 | 455.1 |
| **2008** | 2.2 | 4.8 | 15.8 | 60.3 | 197.3 | 345.6 | 473.8 |
| **2009** | 1.6 | 5.6 | 16.6 | 59.9 | 196.2 | 346.6 | 490.1 |
| **2010** | 1.6 | 4.5 | 16.5 | 60.9 | 199.3 | 346.1 | 473.7 |
| **2011** | 2.3 | 5.8 | 17.0 | 63.9 | 192.7 | 352.5 | 464.4 |
| **2012** | 2.2 | 7.0 | 18.2 | 65.6 | 183.3 | 346.4 | 490.3 |
| **2013** | 2.4 | 7.2 | 17.5 | 64.8 | 172.3 | 327.7 | 462.3 |
| **2014** | 2.8 | 7.6 | 17.5 | 60.1 | 165.9 | 311.7 | 469.6 |
