# Supplementary material for: Colorectal cancer incidence among young adults in England: Trends by anatomical sub-site and deprivation
Source: PLoS One. 2019 Dec 5;14(12):e0225547. doi: 10.1371/journal.pone.0225547 (PMC6894790; doi:10.1371/journal.pone.0225547)
Supplement: S3 Table — (DOCX) [file pone.0225547.s003.docx]

**S3 Table. Annual Percentage Change (APC) in colorectal cancer incidence rates by anatomical sub-site and calendar period of diagnosis in adults aged 20-39 years: England, 1971-2014**

|  | **Persons (N=14,791)** | | | | | **Men (N=7,639)** | | | | | **Women (N=7,152)** | | |  |  |
| --- | --- | --- | --- | --- | --- | --- | --- | --- | --- | --- | --- | --- | --- | --- | --- |
| **Anatomical sub-site** | **Segments** | **APC (%)** | **95% CI** | | | **Segments** | **APC (%)** | **95% CI** | | | **Segments** | **APC (%)** | **95% CI** | | |
| **Right colon** | **1971-1991** | -2.3 | -3.3 | to | -1.4 | **1971-1989** | -2.2 | -3.5 | to | -0.8 | **1971-1991** | -2.7 | -4.1 | to | -1.4 |
|  | **1991-2010** | 5.2 | 4.3 | to | 6.1 | **1989-2010** | 4.5 | 3.4 | to | 5.6 | **1991-2009** | 5.8 | 4.3 | to | 7.4 |
|  | **2010-2014** | 19.4 | 14.5 | to | 24.6 | **2010-2014** | 17.6 | 11.1 | to | 24.4 | **2009-2014** | 20.4 | 13.2 | to | 28.1 |
|  |  |  |  |  |  |  |  |  |  |  |  |  |  |  |  |
| **Left colon** | **1971-1998** | -1.7 | -2.5 | to | -0.8 | **1971-2002** | -0.7 | -1.6 | to | 0.2 | **1971-1997** | -2.3 | -3.1 | to | -1.4 |
|  | **1998-2014** | 5.7 | 4.2 | to | 7.3 | **2002-2014** | 7.3 | 4.0 | to | 10.6 | **1997-2014** | 5.7 | 4.2 | to | 7.2 |
|  |  |  |  |  |  |  |  |  |  |  |  |  |  |  |  |
| **Rectum** | **1971-1990** | -1.6 | -2.7 | to | -0.5 | **1971-1991** | -1.8 | -3.0 | to | -0.7 | **1971-1990** | -1.1 | -2.7 | to | 0.5 |
|  | **1990-2014** | 4.4 | 3.8 | to | 5.1 | **1991-2014** | 4.7 | 4.0 | to | 5.5 | **1990-2014** | 4.1 | 3.2 | to | 5.1 |
|  |  |  |  |  |  |  |  |  |  |  |  |  |  |  |  |
| **Colon, unspecified** | **1971-1996** | 0.2 | -0.7 | to | 1.0 | **1971-1996** | 0.8 | -0.5 | to | 2.1 | **1971-2014** | -1.2 | -1.7 | to | -0.6 |
|  | **1996-2014** | -3.2 | -4.8 | to | -1.6 | **1996-2014** | -3.7 | -6.0 | to | -1.4 |  |  |  |  |  |
