# Supplementary figures and images for: Colorectal cancer incidence among young adults in England: Trends by anatomical sub-site and deprivation
Source: PLoS One. 2019 Dec 5;14(12):e0225547. doi: 10.1371/journal.pone.0225547 (PMC6894790; doi:10.1371/journal.pone.0225547)

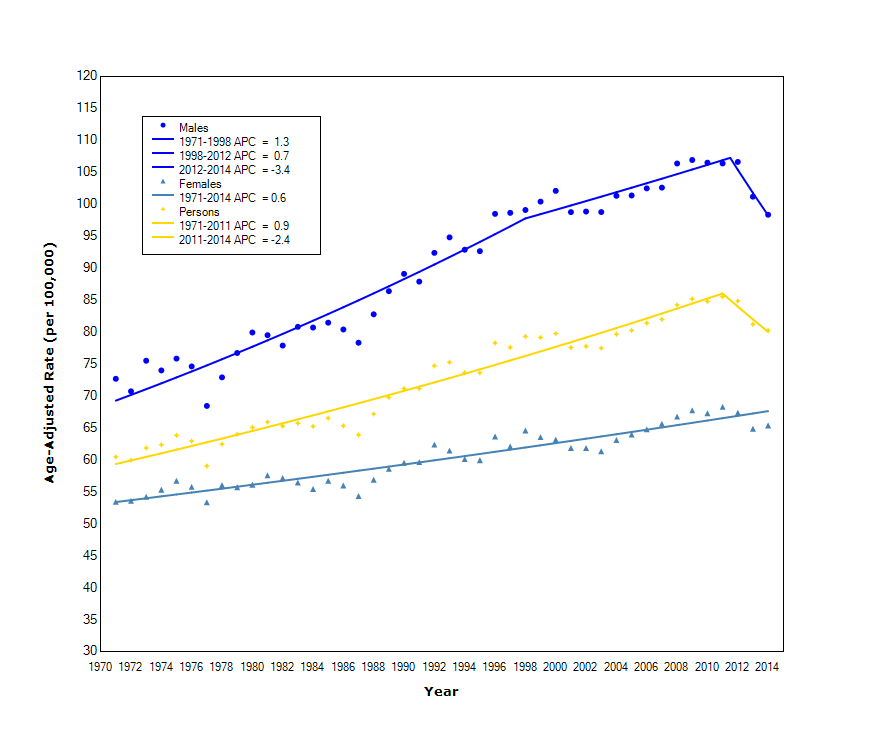

Supplement: S1 Fig — (TIFF) [file pone.0225547.s004.tiff]
